# Pregnancy from a Legal Standpoint—IV

**Published:** 1909-04-10

**Authors:** 


### Medico-Legal Points.

### PREGNANCY FROM A LEGAL STANDPOINT—IV.

Infanticide (*concluded*).

The essential question to be decided by the medical evidence in a case of alleged infanticide is the cause of death. Male children are more liable to die during labour than females, owing to the somewhat larger size of the former, and the greater difficulty of birth. So, too, children die in larger numbers in first than in subsequent labours of the mother: the proportion being one to eleven in the former, and one to thirty-one in the latter. These facts must be taken into account in estimating the probability of death from natural causes. The fœtus may die *in utero* or shortly after birth in consequence of malformations, asphyxia, operation or accidental violence, or from fœtal or maternal disease.

Natural and Accidental Causes of Death.

The medical jurist must be familiar with the many natural and accidental causes of death to which the infant is exposed during birth. The following are amongst the most frequent of these: (1) compression of the umbilical cord before normal respiration is established, if it is not remedied at once, frequently results in the death of the child; (2) suffocation or strangulation may occur from the cord getting round the child's neck—a position of the cord which is found in about one in four deliveries; it may leave a groove or mark suspiciously suggestive of strangulation with a string, but the skin of the neck is not abraded as it most probably would be by a string; (3) in a very protracted labour the child may die from exhaustion or from pressure of the uterus or maternal passages; the child may even exhibit injuries suggestive of violence from the powerful character of the uterine contractions; (4) hæmorrhage from the cord may occasionally be fatal, especially if it has been cut with a sharp instrument and imperfectly ligatured; (5) fractures of the cranial bones are very rare, but are occasionally caused by the use of instruments, or by some abnormal obstructions to delivery; (6) the child may be accidentally asphyxiated owing to the mother being suddenly and unexpectedly delivered while on the seat of a water-closet, and the child being precipitated head down into the pan, where, if it is allowed to remain, it is quickly drowned or suffocated.

A Question of Probabilities.

The last of these natural causes of death is one of the commonest explanations given by the mother, with the addition that she fainted away before she could place the child in safety. In the vast majority of such cases the story is a pure fabrication, but there is no doubt that such rapid delivery has taken place, and has been followed by immediate unconsciousness. The question as to whether the child was accidentally born into the water-closet or was intentionally put there may not be so easy to determine. In the first place, the possibility of the occurrence must be considered. Tardieu (p. 166) considers it scarcely possible for the woman to remain seated during the entire delivery. Either she must be squatting, or she would have to straighten out in order to keep the infant from striking the border of the hole. If she straightened out the infant would be born on the floor, and not in the closet trap, while if the woman was squatting, the child might be born into the closet. Hence, before admitting the possibility of the accident, the exact position of the woman and the arrangements of the closet should be taken into consideration. Again, for such an accident to occur the labour must have progressed much more rapidly than usual, and at a rate that is very exceptional in primiparæ, in whom infanticides are not infrequent. Moreover, for the infant to fall beyond recovery, either the placenta must be born immediately after the child, a very infrequent occurrence, or the cord must be ruptured, which is more likely. In the latter case the end of the cord gives evidence that at least it was not cut, by its irregular end, and the retraction of the blood-vessels, as distinguished from the even end, and comparatively prominent vessels of the cut cord. A child that is immature or badly nourished *in utero* may die from very slight causes which would not affect an ordinarily robust child.

Methods of Suffocation.

Suffocation of the infant may be produced in various ways similar to those in the adult, and also in several ways characteristic of the new-born infant, who can offer no resistance. The face of the infant may be covered with a cloth, pillow, or mattress, none of which will leave any characteristic mark. Or the infant may be buried alive; when, if the dirt has free access to the month and nose, it may be inspired and found in the mouth, pharynx and larynx. The infant may be suffocated by being put in a bureau drawer or in a box, where the asphyxia will follow gradually, and where the signs will also not be characteristic. Similarly, the child's thorax and abdomen may be compressed either with the hands, or by leaving the infant as it is born, between the thighs of the mother, and compressing it there with her thighs. Here, too, the evidence as to the manner in which the suffocation was produced is rarely distinctive.

Perhaps the most usual way for the mother to suffocate the child is to cover the nose and mouth with her hand in her attempt to keep it from crying and so betraying its birth. If the woman succeeds in stopping the cries, she also kills the child. In these cases the signs of the method used are often distinct. There are the marks of the finger-nails of the mother on the face of the child, around the nose and cheeks. These lacerations of the skin are especially likely to occur, as the skin of the child is so slippery from the vernix caseosa, and it is necessary to hold the child fast for five or six minutes to end its attempts at respiration. Owing to the difficulty of holding the child's head, the suffocation is not uncommonly supplemented by strangulation, the mother's hand readily grasping the infant's neck. Here the nail marks are found also on the neck.

To these arguments from the nail marks the mother not infrequently makes the response that the marks are the result of the attempt to help herself in the delivery after the birth of the head by grasping the head with her hands and pulling on it. Such a defence may be admitted if the nail marks are transverse on the neck, but not if they lie in its long axis, as is usually the condition.

### Other Methods of Infanticide.

Burial alive may occur, but more often the body of the child is buried after death, or in a condition of suspended animation at the time of birth. The signs of live burial would be those of suffocation in general, and in addition the presence of the powdered earth in the pharynx and larynx. Bromardel (p. 85) considers that a child may live several hours (four or five) after burial. Hofmann ("Gericht. Med.," p. 800) cites two cases from Bohm, of infants buried just after birth that were dug up alive after having been buried under 25 cms. of earth for eight hours; and still another from Mascha, where the infant was dug up alive after having been under a foot of earth for five hours. Here also may be mentioned the cases of suffocation by exposing the child to noxious vapours, as those of burning charcoal, or sulphur, the exhalations of privies, etc., of which no trace will be found except the odour of the deleterious gases.

Exposure of the child to cold is too slow a method of infanticide for ordinary uses, though, if the exposure is carried only to the point where the child catches bronchitis, and the bronchitis leads to the child's death, then lack of proper care is the indirect cause of death, but leaves no convicting evidence behind. The time that a child can live when abandoned is not definitely settled. If a child is deprived of all nutrition it loses about one hundred grams a day by inanition. Usually at the end of about one week, or when its weight has been reduced to 2,100 grams, it dies. Usually the child is given scant or inappropriate nourishment, and lives a few weeks, leaving no evidence of crime.

### Live Birth and Death by Violence.

It is a fundamental principle laid down by Henke that death by violence is by no means to be inferred from the fact that the child was born alive. Even when marks of death by violence exist, it does not follow that the child was murdered. In the former case it may have perished in consequence of some disease incompatible with its life, or have been suffocated by the caul upon its face, or by its lying in a pool of blood and water, or under a limb of the mother while in a state of exhaustion or unconsciousness; or in consequence of there being no help at hand, or of the unwillingness of the mother to betray her condition, the child may be suffocated, or may perish from exposure to cold, etc. While, says Caspar, we refuse to be imposed upon by the "impudent lies" which women do not hesitate to tell to conceal their guilt, we should not forget that the dangers to new-born children are very numerous, and that, without any criminal intent upon the mother's part, the child may perish from any of the causes just mentioned, from an injury to the head, from constriction of the umbilical cord, or hæmorrhage following its rupture, or from falling into a privy, etc.

Even apparent marks of violence must be cautiously interpreted. It must not be forgotten that many marks of accidental injury are with difficulty to be distinguished from such as are feloniously inflicted. Care should be taken not to confound these with marks which may have been made after death in recovering the body from cess-pools and similar places, or which are merely signs of the voracity of fishes, hogs, rats, etc.

In short, the duty of the medical jurist, called upon to investigate cases like those under consideration, should be to preserve the strictest impartiality, to avoid being biassed by his sympathy with the misfortunes of the accused, upon the one hand, or, on the other, by the abhorrence of her imputed crime, and to endeavour to give its just weight, and no more, to every circumstance which the investigation brings to light.

Among other points, it will be necessary to examine the dimensions of the pelvis of the woman, since this examination may throw some light upon the truth of a defence as to rapid or protracted delivery. Unless an examination of the woman is made within twelve or fifteen days after delivery, no satisfactory evidence can in general be obtained.

If the reputed mother is dead, an order may issue for a post-mortem examination of her body, and the case will present no difficulty; if living, a question may arise as to medical responsibility.

### Refusal of Physical Examination.

It may happen that the woman refuses to submit to a physical examination. An innocent woman is just as likely to refuse permission as one who is guilty; but, if circumstances point to one out of several women in a household, the refusal to permit an examination would of course be interpreted against her. It is only when a woman willingly consents to be examined that a medical man is justified in making an examination. It would, however, be proper in such a case to give her the warning which every magistrate and coroner is bound to give to any woman charged with murder, before requiring an answer to a question which may be used in evidence against her at the subsequent trial.

If a medical man compels a suspected woman, unwillingly, or under duress, to submit to a physical examination, he is forcibly compelling a woman accused of murder to produce positive proof of her guilt. There is no statute which authorises such a proceeding. Any coroner issuing such an order to a medical man would be acting *ultra vires*, and any medical man obeying it would render himself liable to damages.

